# Severe Aqueous-Deficient Dry Eye Following Herpes Zoster Ophthalmicus With Periocular Inflammation: A Case Series

**DOI:** 10.7759/cureus.90569

**Published:** 2025-08-20

**Authors:** Rina Kanaya, Yoshiaki Tagawa, Kenichi Namba, Mayuko Kimura, Susumu Ishida

**Affiliations:** 1 Department of Ophthalmology, Hokkaido University, Sapporo, JPN; 2 Department of Ophthalmology, Health Sciences University of Hokkaido, Sapporo, JPN; 3 Department of Ophthalmology, Tokeidai Memorial Hospital, Sapporo, JPN

**Keywords:** aqueous-deficient dry eye, herpes zoster ophthalmicus, neuropathic ocular pain, neurotrophic keratopathy, periocular inflammation, varicella-zoster virus

## Abstract

This series aimed to describe the clinical characteristics and course of severe aqueous-deficient dry eye (ADDE), a condition characterized by tear deficiency and reduced corneal sensation, following ophthalmic herpes zoster infection with periocular inflammation. Four cases of ADDE that developed after ophthalmic herpes zoster infection were retrospectively examined. Clinical data, including disease course, medical history, imaging findings, tear volume assessment, corneal sensitivity testing, and treatment outcomes, were collected. Our patients exhibited severe tear deficiency, significant corneal hypoesthesia, and serious corneal complications, accompanied by persistent ocular pain despite reduced corneal sensation. Imaging studies revealed dacryoadenitis in two cases and suggested severe periocular inflammation in all cases. Persistent superficial punctate keratopathy (SPK) was observed in all patients, with complications including corneal ulcers, perforation, persistent epithelial defects, and filamentary keratitis. Neuropathic pain was believed to contribute to persistent ocular pain. Treatment with topical rebamipide, autologous serum eye drops, punctal plugs, and eyelid closure effectively managed corneal epithelial damage and associated complications. Herpes zoster-induced dacryoadenitis and corneal sensory nerve damage probably contributed to ADDE pathogenesis. Given the potential for severe corneal complications, careful long-term monitoring is crucial in these patients.

## Introduction

Herpes zoster ophthalmicus (HZO), which occurs in 7.9% of herpes zoster cases, affects the first branch of the trigeminal nerve (TG)[1]. Although keratitis and iridocyclitis are well-documented ocular complications [2], severe inflammation of the periorbital tissues, including dacryoadenitis, has also been reported, albeit less frequently [3,4]. Dry eye disease (DED) affects 11% of the global population and is classified into aqueous-deficient, evaporative, and mixed-type [5,6]. DED is a condition characterized by ocular discomfort and visual dysfunction [7]. Aqueous-deficient dry eye (ADDE), exemplified by dry eye associated with Sjögren's syndrome, is considered the most typical form of dry eye resulting from insufficient tear production.

ADDE is characterized by reduced tear secretion, primarily caused by lacrimal gland dysfunction or autonomic nerve abnormalities [8]. Decreased corneal sensitivity [9] and neuropathic pain are common features in patients with ADDE [10,11]. In contrast, neurotrophic keratopathy does not cause ocular pain but can lead to severe corneal complications [12]. HZO can lead to neurotrophic keratopathy due to corneal nerve impairment [13]. On the ocular surface, afferent input from the corneal nerves triggers reflex tear secretion and blinking. Therefore, corneal nerve damage caused by HZO may result in DED due to reduced tear production and decreased blink frequency [14]. Although corneal sensory deficits are common in patients with HZO, severe ocular pain due to neuropathic pain has also been reported [13].　

This study aimed to describe four cases of severe ADDE following dacryoadenitis or orbital inflammation associated with HZO. These cases were complicated by severe ocular pain and corneal pathology. To the best of our knowledge, this is the first report of severe ADDE cases after HZO with periocular inflammation.

## Case presentation

Case 1

A female in her 60s presented with right ocular pain, swelling of the right upper eyelid, and vesicular eruptions in the distribution of the first and second branches of the TG territory. Despite treatment with acyclovir ophthalmic ointment for keratitis, ocular pain persisted, leading to admission. Her medical history included Meniere’s disease, arrhythmias, and anxiety disorders. She had no previous history of ophthalmic disease or ocular surgery.

**Figure 1:**
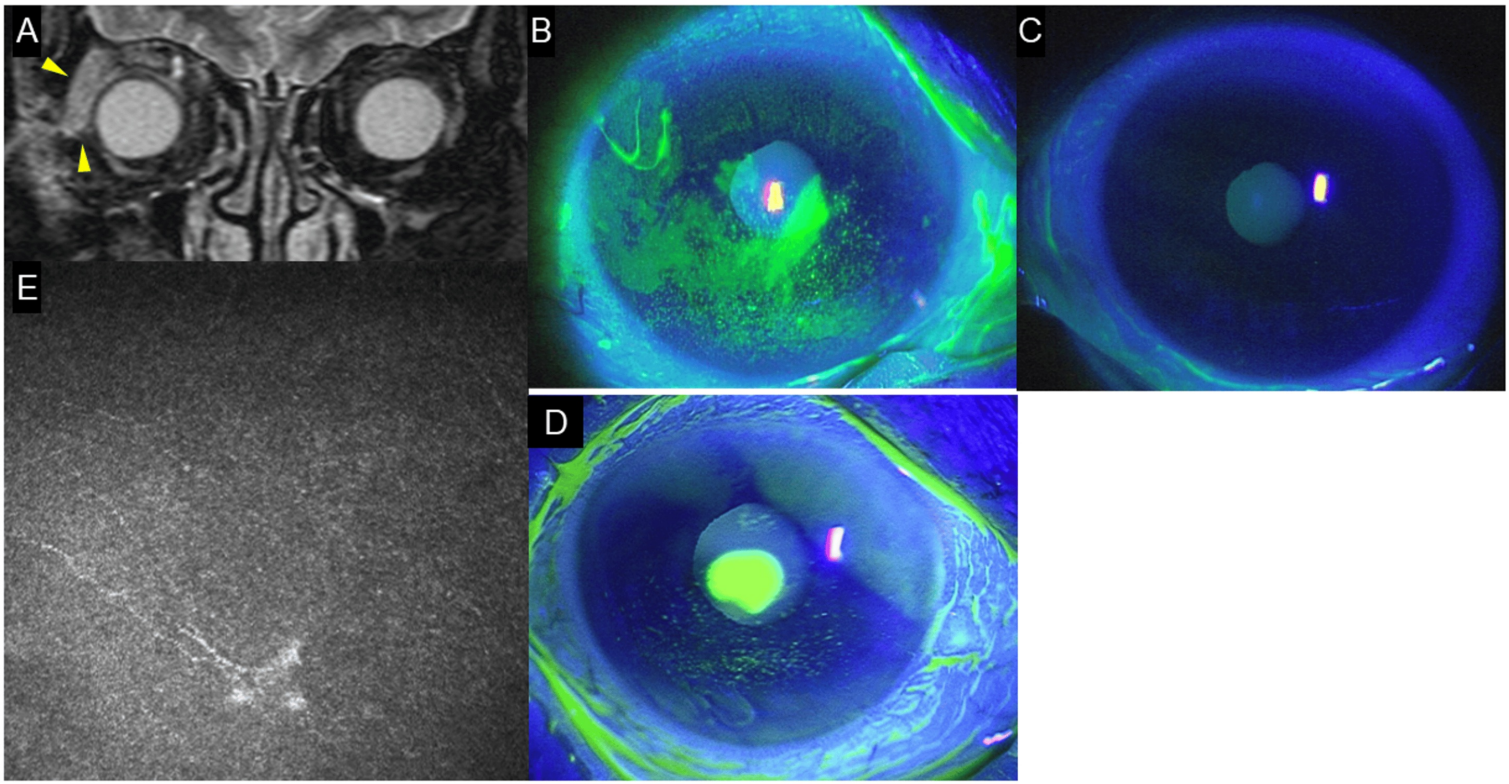
Case 1 - Images showing neuroparalytic keratitis after HZO with dacryoadenitis (A) Axial coronal MRI of the forehead. The right lacrimal gland is swollen (yellow arrowhead). (B) Right anterior segment photograph with fluorescein staining. After orbital inflammation subsided, epithelial damage appeared on the right cornea and conjunctiva. (C) Left anterior segment photograph with fluorescein staining. No epithelial damage is observed on the left cornea and conjunctiva. Similarly, no epithelial defects were observed on the unaffected eye in the other three cases. (D) Right anterior segment photograph with fluorescein staining. Following the insertion of punctal plugs and discontinuation of rebamipide eye drops, a circular corneal ulcer appeared in the paracentral area of the cornea, with little conjunctival injection or corneal infiltration. The ulcer was diagnosed as being associated with neurotrophic keratopathy. (E) Confocal microscopy image of the central cornea. Corneal nerves are visible but significantly reduced HZO: herpes zoster ophthalmicus; MRI: magnetic resonance imaging

On initial examination, her visual acuity was 20/30 (right) and 20/13 (left), with normal intraocular pressure measured using Goldmann applanation tonometry. Keratitis and eyelid swelling resolved; however, the patient exhibited severe ptosis of the eyelid, restricted eye movements in all directions, and mild optic disc hyperemia. The position of the eyeball was inwardly deviated, but no obvious proptosis was observed. No significant abnormalities were observed in the left eye. MRI revealed enlargement of the right lacrimal gland and extraocular muscles (Figure 1A). High levels of varicella-zoster virus IgM were detected in the cerebrospinal fluid, confirming HZO diagnosis.

Famciclovir 500 mg was administered orally three times daily for two weeks, resulting in improved ocular motility and reduced lacrimal gland swelling. Subsequently, superficial punctate keratopathy (SPK) developed (Figures 1B, 1C), and the Schirmer test demonstrated a marked reduction in tear production in the right eye two months after the initial visit (Table 1; right: 2 mm, left: 29 mm). Epithelial damage was attributed to tear deficiency, and the administration of rebamipide eye drops improved SPK temporarily. Four months after the initial visit, due to worsening dry eye, epithelial damage was exacerbated in the lower cornea, and an ulcer formed in the inferotemporal region, but improved to some extent with additional steroid eye drops. Due to persistent epithelial damage, five months after the initial visit, punctal plugs were inserted into the upper and lower puncta of the right eye, significantly improving SPK and allowing rebamipide discontinuation. Two weeks after that, due to neurotrophic keratopathy, a central corneal ulcer developed without conjunctival hyperemia or stromal infiltration (Figure 1D). Corneal sensitivity assessed using a Cochet-Bonnet esthesiometer revealed reduced sensitivity (20 mm; Table 1). The central corneal ulcer was suspected to be attributed to neurotrophic keratopathy, and four daily doses of rebamipide eye drops were reintroduced to promote ulcer healing. Confocal microscopy revealed a reduction in the number of corneal nerve fibers (Figure 1E). However, deep ocular pain persisted. The symptoms were somewhat alleviated by continuing to take 25 mg of pregabalin, a medication for neuropathic pain, twice daily for several months.

**Table 1:** Summary of the clinical information of the patients ^*^The corneal sensitivity in Case 3 indicates whether or not the sensation of touch was present during the Schirmer test The Schirmer test was performed two months after the initial visit in Cases 1 and 2, three years after the onset of HZO in Case 3, and nine years after the onset of HZO in Case 4 HZO: herpes zoster ophthalmicus; SLE: systemic lupus erythematosus; VZV: varicella-zoster virus

	Age, years	Sex	Past medical history	Findings for VZV	Schirmer test		Corneal sensitivity	
					Affected eye	Fellow eye	Affected eye	Fellow eye
Case 1	60s	F	Meniere's disease	V1, 2 skin eruption	2 mm	29 mm	20 mm	60 mm
			Arrhythmia	Dacryoadenitis				
			Anxiety disorder	Extraocular myositis				
Case 2	60s	F	Colon cancer	V1 skin eruption	4 mm	16 mm	5 mm	40 mm
				Keratitis, iridocyclitis				
			Metastasis to the lymph node	Dacryoadenitis				
			During chemical therapy	Oculomotor neuritis				
				Optic neuritis (probably)				
Case 3	60s	F	Dialysis	V1 skin eruption	3 mm	18 mm	No^*^	Yes^*^
				Episcleritis				
			Lagophthalmia	Oculomotor neuritis				
				Optic neuritis (probably)				
Case 4	30s	F	SLE	V1-3 skin eruption	5 mm	35 mm	5 mm	60 mm
			Dialysis	Meningoencephalitis				

Case 2

A female in her 60s with a history of lymph node and liver metastases from colorectal cancer, undergoing chemotherapy, presented with a rash on the left side of her forehead, consistent with the distribution of the first branch of the TG. This rash was diagnosed as HZO, accompanied by conjunctival hyperemia in the left eye. She was initially treated with oral valacyclovir for two weeks at a general medical clinic. Subsequently, she developed left ocular pain, ptosis, and corneal erosion and was referred to our hospital. She had no previous history of ophthalmic disease or ocular surgery.

**Figure 2:**
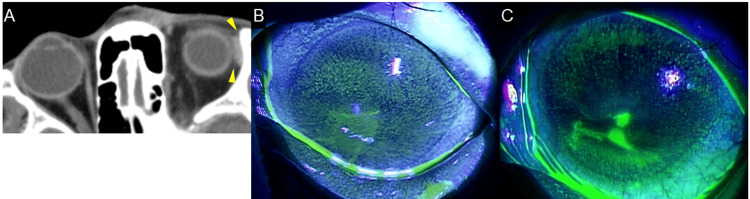
Case 2 - Images showing a prolonged epithelial defect after HZO with dacryoadenitis (A) Axial horizontal contrast-enhanced CT of the orbit. The left lacrimal gland is swollen (yellow arrowhead). (B) Left anterior segment photograph with fluorescein staining. Epithelial damage is observed on the left cornea and conjunctiva. (C) Erosion is visible on the left cornea, extending from the central area downward CT: computed tomography; HZO: herpes zoster ophthalmicus

Her visual acuity at the initial visit was 20/15 (right) and 20/70 (left). Intraocular pressure measured using Goldmann applanation tonometry was 9 and 24 mmHg in the right and left eye, respectively. Clinical examination of the left eye revealed ptosis, limited adduction, a slightly delayed pupillary light response, and paralytic mydriasis. Geographic corneal erosion and stromal edema were also observed. No abnormalities were observed in the right eye. Orbital contrast-enhanced CT revealed the left lacrimal gland enlargement and enhancement surrounding the left oculomotor and optic nerves (Figure 2A).

The patient was diagnosed with keratitis associated with HZO and treated with acyclovir ophthalmic ointment and topical steroids. Even though systemic steroid therapy was recommended, she declined. Although corneal erosion improved, SPK persisted (Figure 2B). We confirmed markedly reduced tear production and corneal sensitivity two months after the initial visit (Table 1). The patient reported persistent, intense ocular pain. Orbital inflammation, including lacrimal gland inflammation, may also have contributed to the ocular pain. Rebamipide eye drops improved SPK; however, four months later, corneal erosion recurred (Figure 2C). The patient was diagnosed with a persistent epithelial defect associated with neurotrophic keratopathy. She was instructed to apply an ophthalmic ointment, wear an eye patch, and keep her eyelids closed; the ulcer subsequently improved.

Case 3

A female in her 60s with a history of bilateral retinitis pigmentosa and baseline visual acuity of 20/500 in both eyes presented with HZO in the distribution of the left first branch of the TG, with a rash on her left forehead. She was treated with oral valacyclovir but, subsequently, developed left ocular pain and difficulty opening her eyelids, prompting her to visit our hospital. Her medical history included angina pectoris, hemodialysis for chronic renal failure, and mild left lagophthalmos secondary to facial nerve palsy following left cerebellar tumor resection. However, no corneal epithelial damage had been observed before this presentation. She had no previous history of ocular surgery.

**Figure 3:**
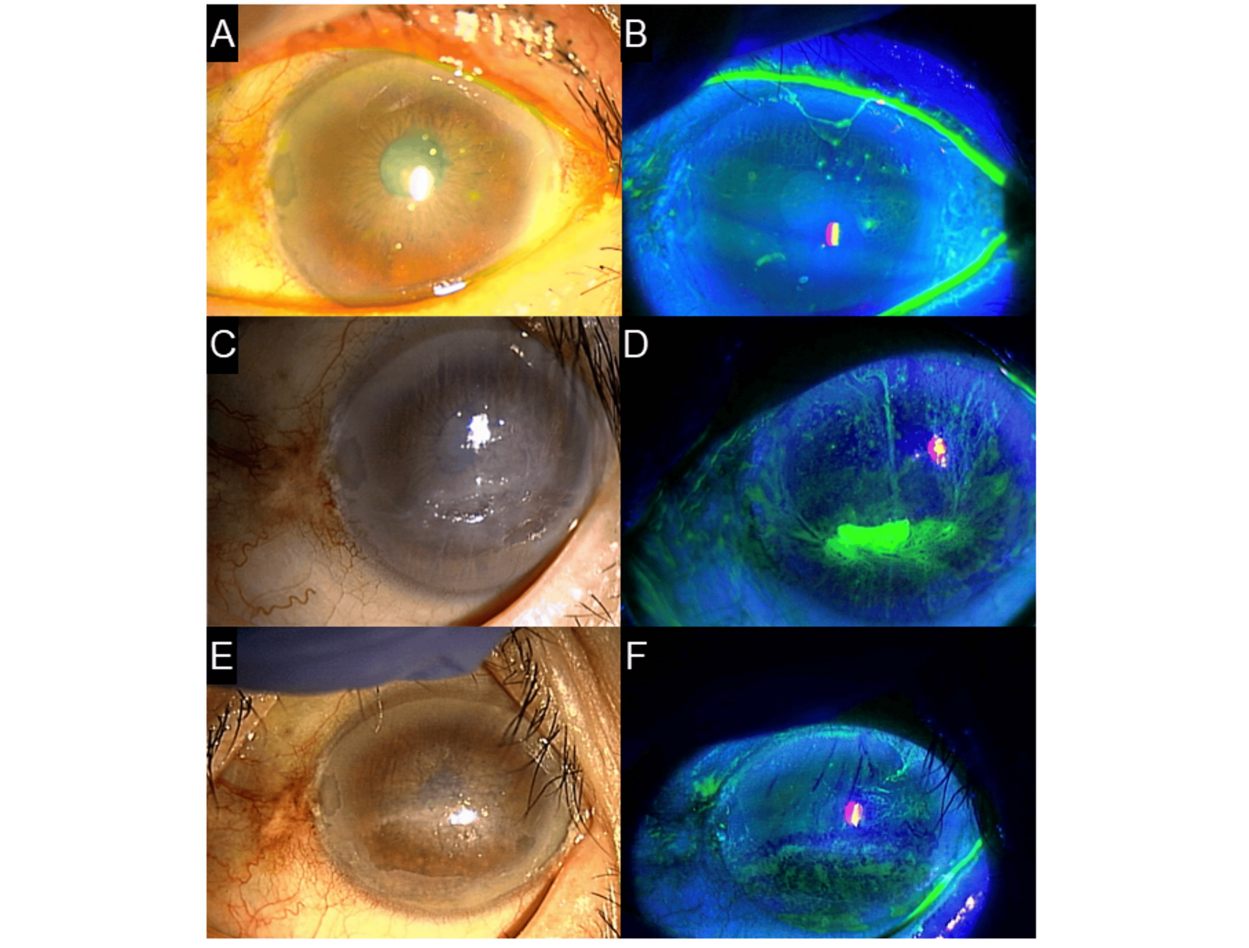
Case 3 - Images showing a corneal erosion with filamentary keratitis after HZO with periocular inflammation (A, B) Left anterior segment photographs before the emergence of keratitis. No corneal epithelial defects were noted except for a little band keratopathy. (C, D) Left anterior segment photographs after the emergence of keratitis. Erosion is observed slightly below the center of the cornea, accompanied by filamentary keratitis in the surrounding area. (E, F) Left anterior segment photographs after the treatment of keratitis. Corneal opacity remains, but epithelial damage has disappeared HZO: herpes zoster ophthalmicus

On initial examination, her visual acuity was 20/400 (right) and 20/2000 (left), with normal intraocular pressure measured using Goldmann applanation tonometry. Swelling and ptosis of the left upper eyelid were observed, along with restrictions in upward, downward, and inward movements of the left eye. No corneal epithelial defects were noted except for a little band keratopathy (Figures 3A, 3B). The anterior segment of the right eye appeared normal, with no new abnormalities in the fundus.

The patient was diagnosed with a left oculomotor nerve palsy secondary to HZO. However, as one month had elapsed since symptom onset, systemic steroid therapy was not initiated. Over the following three months, eruption and eye movement gradually improved. Conversely, the left eye SPK emerged. Eyelid closure therapy was attempted but proved ineffective. Subsequently, corneal erosion and filamentary keratitis developed (Figures 3C, 3D). Treatment with rebamipide and autologous serum eye drops gradually improved erosion and ocular pain (Figures 3E, 3F). Although topical anesthesia was administered in the examination room, the pain did not improve sufficiently. Therefore, not only pain due to ocular surface disorders but also neuropathic pain seemed to be involved. Since autologous serum eye drops also affect improving neuropathic pain, both ocular surface disorders and ocular pain seemed to improve. However, reduced tear production was observed three years after the onset of HZO (Table 1). During the Schirmer test, the patient reported discomfort from the test strip in the right eye but no sensation in the left eye, indicating a marked reduction in corneal sensitivity in the left eye.

Case 4

A female in her 30s presented with a history of HZO involving the right ophthalmic to mandibular branches of the TG, which had developed nine years earlier. This episode was complicated by meningoencephalitis, requiring intravenous antiviral therapy. She had experienced chronic ocular pain for nine years since the onset of HZO and was referred to our hospital. Her medical history included systemic lupus erythematosus (SLE) and hemodialysis for chronic renal failure. She had no previous history of ocular surgery.

At the initial visit, her visual acuity was 20/25 in the right eye and 20/13 in the left eye, with normal intraocular pressure measured using Goldmann applanation tonometry. Examination revealed significant SPK in the right eye, whereas the posterior segments of the right and entire left eyes were unremarkable. We observed marked reductions in tear production and corneal sensitivity nine years after the onset of HZO (Table 1).

**Figure 4:**
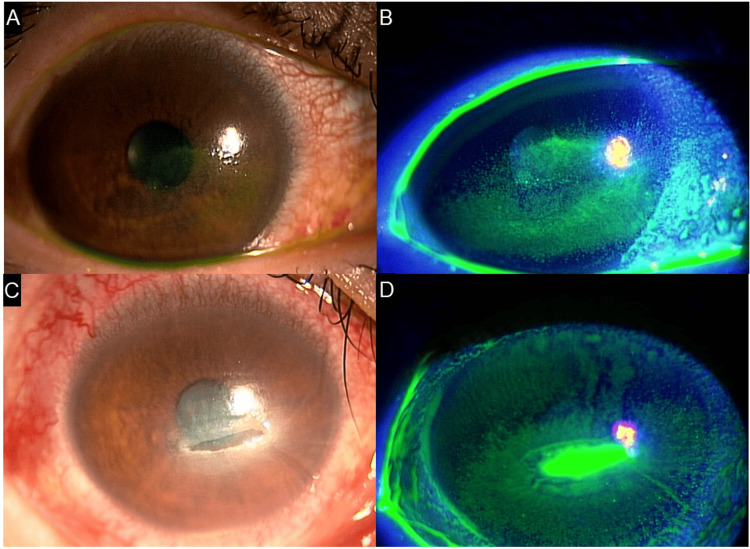
Case 4 - Images showing SPK or corneal erosion for nine years since the onset of HZO (A, B) Right anterior segment photographs. Epithelial damage is present on the cornea and conjunctiva. (C, D) Right anterior segment photographs. Erosion is noted slightly below the central cornea HZO: herpes zoster ophthalmicus; SPK: superficial punctate keratopathy

Despite treatment with multiple topical medications, such as hyaluronic acid, rebamipide, diquafosol, and steroids, and punctal plug insertion, no significant improvement in the SPK was observed (Figures 4A, 4B). Two years after the initial visit, a corneal ulcer developed in the paracentral region, progressing to a perforation (Figures 4C, 4D). Rapid epithelialization of the ulcer was observed after topical steroid administration.

## Discussion

The four patients in this series developed severe ADDE with marked tear reduction and corneal sensory loss following HZO complicated by dacryoadenitis or severe inflammation of the periorbital tissues. To the best of our knowledge, this is the first study to document severe ADDE after HZO with periocular inflammation. These HZO cases were characterized by severe inflammation of the lacrimal gland, which is innervated by the first branch of TG, and periorbital tissues, followed by the onset of severe ADDE with associated ocular pain after inflammation resolution. Shingles’ risk factors include advanced age, immunodeficiency, chronic cardiovascular or renal disease, malignant tumors, SLE, and poor mental health [15,16]. All patients had multiple overlapping risk factors that potentially contributed to the severe inflammation. Although dacryoadenitis is a rare HZO complication, it is often accompanied by extensive periorbital tissue inflammation [3,4,17,18].

Severe periorbital inflammation in Cases 3 and 4 suggests the possibility of associated lacrimal gland or autonomic nerve inflammation. In addition, all patients exhibited reduced corneal sensitivity. Consequently, severe tear reduction probably resulted from a combination of lacrimal gland dysfunction owing to dacryoadenitis and impaired reflex tear secretion caused by trigeminal and autonomic nerve damage. Despite reduced corneal sensation, severe ocular pain was observed, strongly suggesting the involvement of neuropathic pain mechanisms. We hypothesize that severe ocular pain that persists or is not alleviated by topical anesthesia may be associated with central sensitization due to neuropathic pain of the ocular surface and inflammation of the periocular tissues.

All four patients exhibited severe corneal complications in the context of marked tear deficiency and neurotrophic keratopathy. Persistent SPK was observed in all cases. Recurrent corneal ulcers were reported in Case 1, persistent corneal erosion in Cases 2 and 3, and corneal ulceration with perforation in Case 4. These findings were attributed to the combination of severe neurotrophic keratopathy and pronounced ADDE. Rebamipide, which has been reported to be effective against diabetic neurotrophic keratopathy [19], demonstrated efficacy in these cases. Rebamipide was originally developed and used in Japan over 40 years ago as a therapeutic agent for peptic ulcers. In 2013, it was approved in Japan as a treatment for DED, specifically targeting improvement of the ocular surface mucosa [20]. Rebamipide is known for its potent anti-inflammatory properties and epithelial healing effects. In our cases, it appeared to exert partial efficacy in managing neurotrophic keratopathy and neuropathic ocular pain. However, since it was administered in combination with other medications, including autologous serum eye drops and pregabalin, further investigation is required to validate its specific therapeutic effects.

## Conclusions

We reported four cases of severe ADDE following HZO with dacryoadenitis or periocular inflammation, posing a risk of corneal sensory deficits and serious corneal complications. Lacrimal gland impairment and corneal sensory nerve damage likely contributed to the pathogenesis of ADDE. Careful long-term follow-up is essential for effective monitoring and management of these patients.
